# Ureteroscopic Stone Extraction in Cross-Fused Renal Ectopia

**DOI:** 10.1089/cren.2018.0063

**Published:** 2018-12-17

**Authors:** Amir Toussi, Timothy Boswell, Aaron Potretzke

**Affiliations:** Department of Urology, Mayo Clinic, Rochester, Minnesota.

**Keywords:** cross-fused renal ectopia, ureteroscopy, ureteral stone

## Abstract

***Background:*** Urinary stone disease in cross-fused renal ectopia is rare and the aberrant anatomy poses challenges to treatment options. The available literature on treatment modalities remains limited. In this study, we present a case of ureteral stone in a cross-fused renal ectopia managed through retrograde approach.

***Case Presentation:*** We present a case of a 69-year-old woman with an obstructing ureteral stone in a cross-fused renal ectopia managed with ureteroscopic stone extraction. With the use of a ureteral access sheath, holmium laser, and Nitonol basket, the stone was fragmented and removed through retrograde access. The stone composition was 100% calcium oxalate monohydrate and her 24-hour urine collection was only significant for low volume.

***Conclusion:*** With special modifications and attention to the individual patient's anatomy, retrograde approach with the use of an access sheath is safe and effective for treatment of ureteral stones in patients with cross-fused renal ectopia.

## Introduction and Background

Cross-fused renal ectopia is the second most common renal fusion anomaly after horseshoe kidney, with an incidence of 1:1000. Stone formation in these patients is rare and the majority remain asymptomatic throughout their lifetime; however, associated factors such as kidney malrotation, anomalous renal vasculature, relative urinary stasis, and metabolic abnormalities make such patients prone to urinary calculi.^1^ Because of the aberrant anatomy, management of stones in these patients has previously been heterogeneous and dependent on surgeon preference and experience. In this study, we present a case of ureteral stone in a patient with cross-fused renal ectopia managed through retrograde approach.

## Presentation of Case

**FIG. 1.**
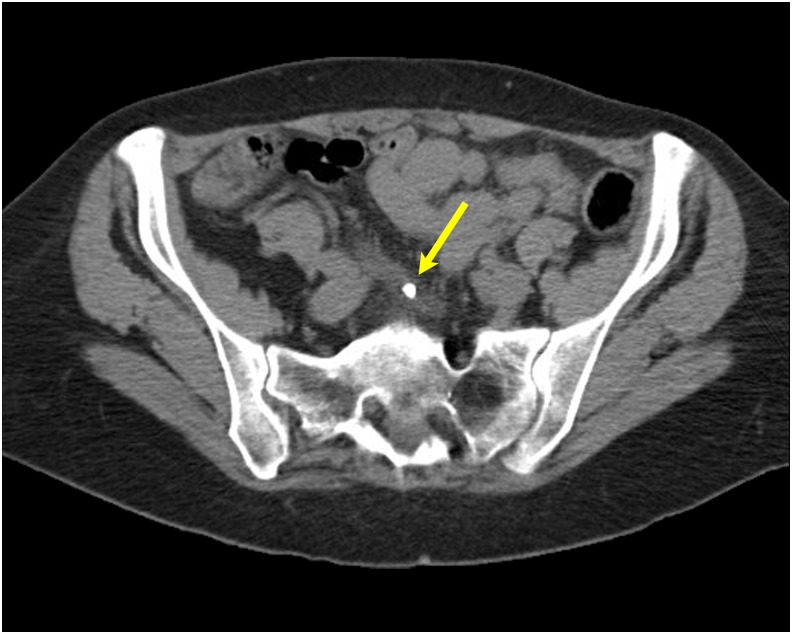
CT scan with stone in the mid ureter shown by the *yellow arrow*. CT, computed tomography.

Our patient was a 69-year-old woman who was referred to the clinic after presenting to the emergency department with right flank and pelvic pain. CT scan showed a 7-mm mid ureteral stone in a right-sided cross-fused renal ectopia (Fig. 1). She was hemodynamically stable. She exhibited right lower quadrant and flank pain on examination. Her white blood count was 11.7, creatinine was 0.6, and urinalysis showed >100 RBC/hpf. She was consented for ureteroscopic stone extraction with stent placement.

**FIG. 2.**
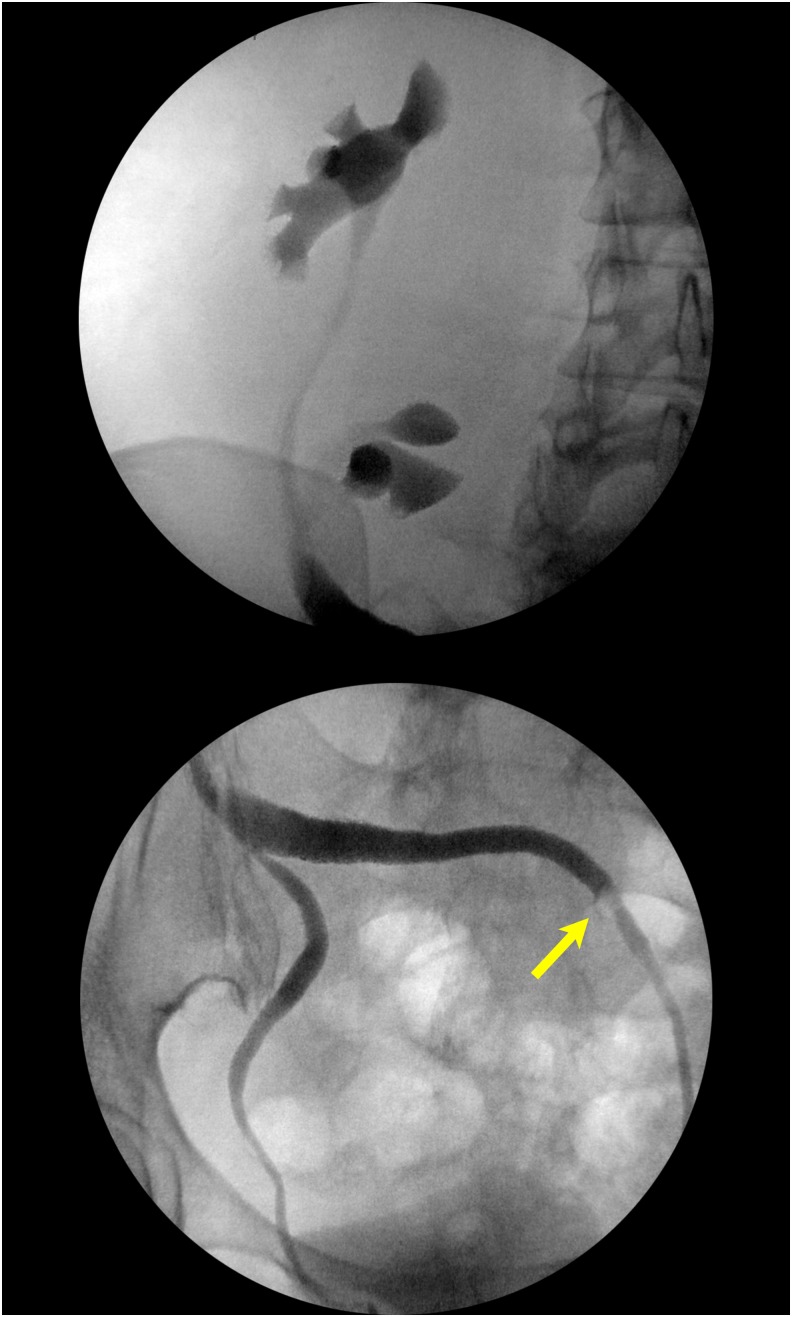
Bilateral retrograde pyelogram depicting the urinary system of the right-sided cross-fused renal ectopia. *Arrow* is pointing to the location of the stone in the left mid ureter.

**FIG. 3.**
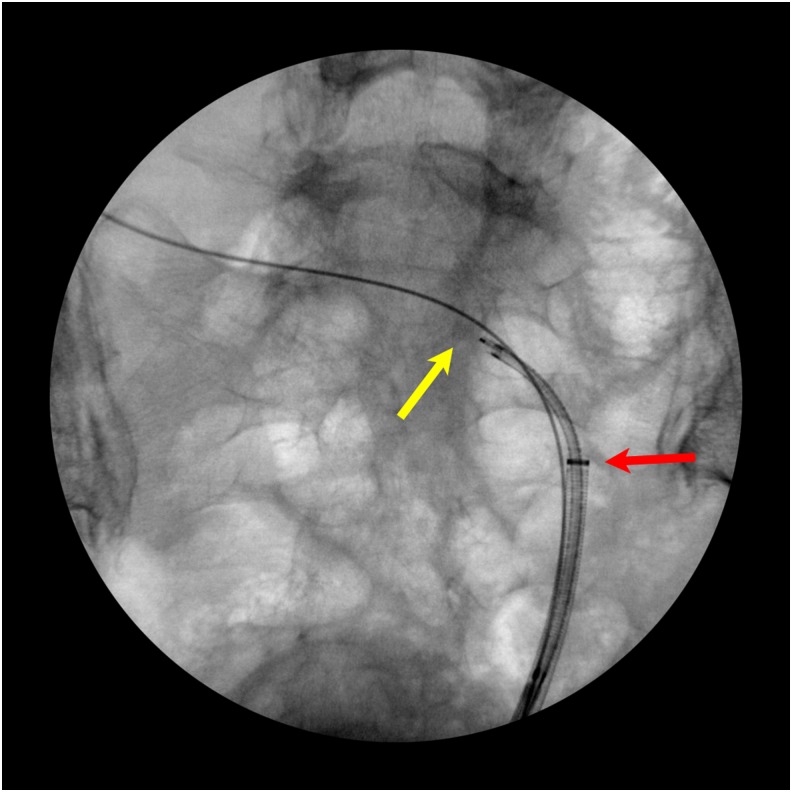
Flexible ureteroscopy was used to remove the stone. *Yellow arrow* points to the stone and *red arrow* points to the position of the access sheath tip.

**FIG. 4.**
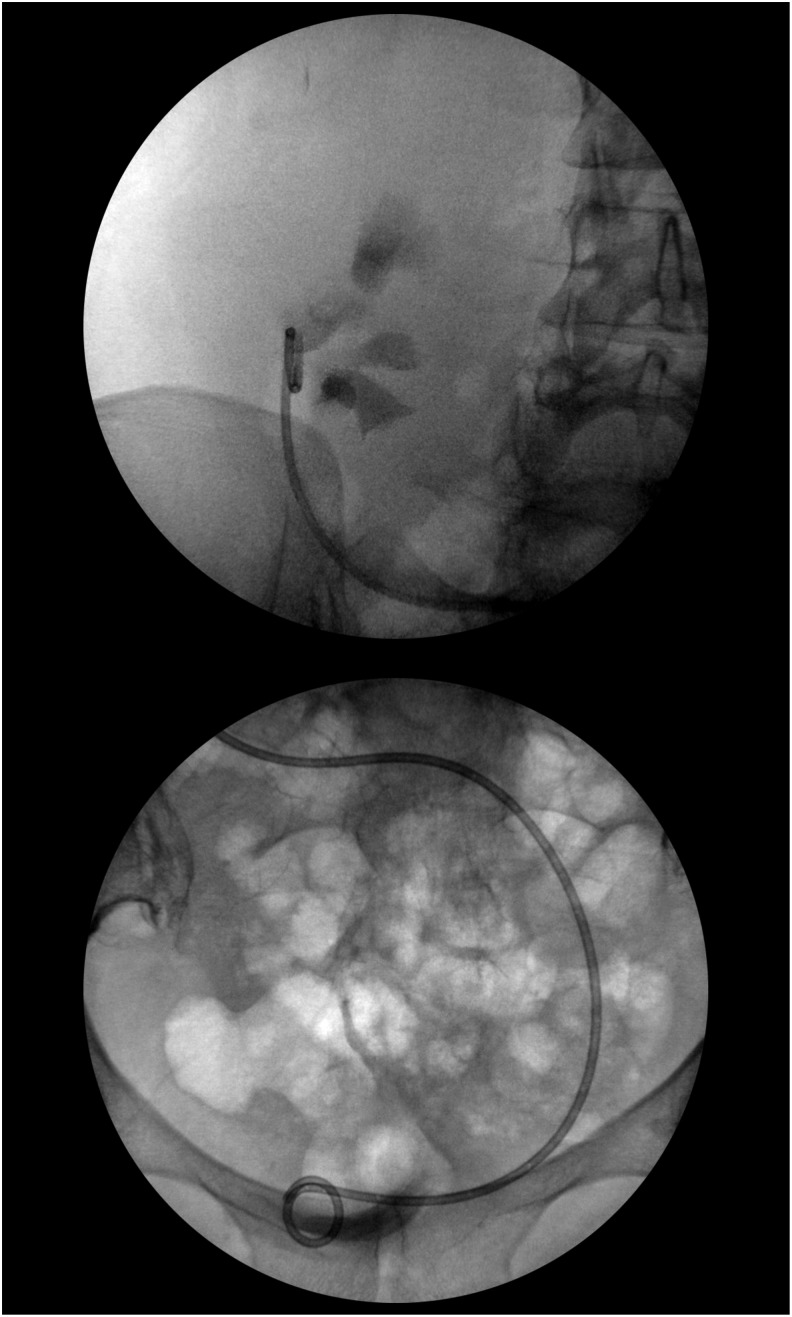
Double-J ureteral stent placement.

During the operation, cystoscopy showed bilateral single orthotopic ureteral orifices. Bilateral retrograde pyelogram was performed, showing a right-sided cross-fused renal ectopia. The stone was seen in the left mid ureter (Fig. 2). After placement of 0.035 inch sensor tip working and safety wire, an 11/13F ureteral access sheath was advanced. Care was taken not to advance it past the acute angulation of the ureter as it crossed the pelvis to the right side (Fig. 3). Using the Olympus (Tokyo, Japan) P6 flexible ureteroscope, the stone was fragmented with the 200-μg holmium laser. The stone fragments were then extracted with the 1.9F Nitinol basket (Cook Medical, Bloomington, IN). The entire upper tract urinary system was inspected and found to be free of stones or injury. Then using a 5F ureteral access catheter, the length of the ureter was measured at 26 cm, thus a 7F × 26 cm Double-J ureteral stent was placed on a dangle (Fig. 4). The patient removed the stent 5 days later without complications.

She underwent a renal and bladder ultrasonography and kidney, ureter, and bladder radiograph 6 weeks after the procedure. These showed no residual stones or hydronephrosis. Stone composition was 100% calcium oxalate monohydrate. Her 24-hour urine collection was significant for low urine volume (1300 cc). The remainder of her study was within the normal range, including urinary calcium, oxalate, uric acid, and citrate levels. She was counselled on appropriate dietary modifications.

## Discussion and Literature Review

Aberrant renal anomaly such as cross-fused renal ectopia poses a challenge to management of ureteral stones. Previous case reports have described a diverse range of management modalities, including open, laparoscopic, percutaneous, and retrograde approaches.^2,3^ It is important to note that these reports included patients with intrarenal stones only, and to our knowledge this is the first case report of a ureteral stone in cross-fused renal ectopia.

With the advances in endourology technology, retrograde access with the use of access sheath can be used to treat stones. The use of an access sheath allows for expeditious passes through the ureter and the use of continuous pressure irrigation, which ultimately decreases operative times and improves stone-free rate. However, risks specific to ureteral access sheaths exist and include the potential for ureteral injury. In as many as 16% of cases ureteral access sheath insertion may fail or result in an injury because of ureteral kinking or aberrant anatomy.^4^

Certain considerations must be taken when using access sheaths in patients with aberrant anatomy. In this special case, extra care was taken while advancing the access sheath. The left ureter traverses a cross-body course to reach the right-sided kidney, which results in an acute angle at the level of the mid ureter (Fig. 3). Therefore, the access sheath was not passed beyond this point to avoid inadvertent ureteral perforation. Also, an 11/13F access sheath was used, which is more flexible than the larger size access sheaths. Furthermore, liberal but pulsed fluoroscopy should be used when passing the sheath to ensure progression. In our opinion, it is important to use a stent at the conclusion of the case since the meandering course of the ureter may result in higher risk of symptoms from ureteral edema.

## Conclusion

With special modifications and attention to the individual patient's anatomy, retrograde approach with the use of an access sheath is safe and effective for treatment of ureteral stones in patients with cross-fused renal ectopia.

## Abbreviation Used

CT: computed tomography
